# Purinergic signalling in bone

**DOI:** 10.3389/fendo.2012.00116

**Published:** 2012-09-19

**Authors:** Robin M. H. Rumney, Ning Wang, Ankita Agrawal, Alison Gartland

**Affiliations:** Department of Human Metabolism, The Mellanby Centre for Bone Research, The University of SheffieldSheffield, UK

**Keywords:** ATP, purinergic signaling, P2Y, P2X, osteoblast, osteoclast, bone, osteoporosis

## Abstract

Purinergic signaling in bone was first proposed in the early 1990s with the observation that extracellular ATP could modulate events crucial to the normal functioning of bone cells. Since then the expression of nearly all the P2Y and P2X receptors by osteoblasts and osteoclasts has been reported, mediating multiple processes including cell proliferation, differentiation, function, and death. This review will highlight the most recent developments in the field of purinergic signaling in bone, with a special emphasis on recent work resulting from the European Framework 7 funded collaboration ATPBone, as well as Arthritis Research UK and Bone Research Society supported projects.

## INTRODUCTION

Purinergic signaling is the most primitive and ubiquitous cell-to-cell signaling system that exists (Burnstock and Verkhratsky, 2012a). It involves the energy molecule adenosine triphosphate (ATP) and its breakdown products being used outside of the cell to bind specific receptors called purinoceptors which then direct the action and fate of cells. Just as every living organism uses ATP for energy, nearly every cell will express purinoceptors and use purinergic signaling to control cellular functions (for a recent review, see Burnstock and Verkhratsky, 2012b).

In the context of bone, purines and pyrimidines are present in the extracellular milieu, either as a consequence of lytic or controlled release. Upon binding specific cell surface purinoceptors expressed by the different cell types in bone, intracellular calcium signaling cascades are triggered to direct the fate of bone cells and ultimately control bone homeostasis. Regulation of purinergic signaling in bone involves the co-ordinated actions of (1) controlled release of purines and pyrimidines from bone cells; (2) the expression and activity of enzymes on the surface of bone cells that can breakdown or interconvert ATP and other nucleotides; (3) purinoceptor expression by bone cells which is tightly controlled in a temporal and spatial manner.

Purinergic signaling in bone was first proposed in the early 1990s with the observation that extracellular ATP could raise intracellular calcium and induce secondary messenger activation – events crucial to the normal functioning of osteoblasts (Kumagai et al., 1991; Schofl et al., 1992). Since then it has been reported that osteoblasts and osteoclasts (of one species/type or another) express nearly all of the P2Y and P2X receptors, mediating multiple cellular processes and providing a mechanism to integrate local (i.e., ATP) and systemic (i.e., PTH) signaling to control bone turnover. For an extensive historical reviews of purinergic signaling in bone, see Grol et al. (2009), Orriss et al. (2010), Reyes et al. (2011), Gartland (2012), Gartland et al. (2012a), and Bowler et al. (2001).

**FIGURE 1:**
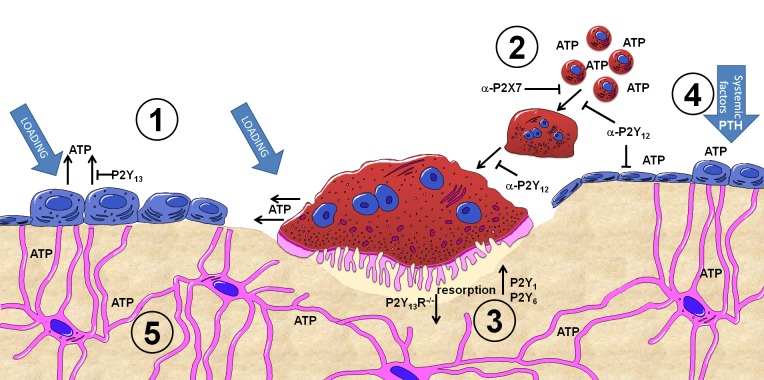
**Summary of purinergic signaling in bone.** P2Y and P2X receptor subtypes are expressed by most bone cell types and are activated by extracellular nucleotides present in the microenvironment. **(1)** ATP release from osteoblasts and osteoclasts occurs both constitutively and as a consequence of mechanical loading, with the concentration of ATP released varying in a time, direction and strain dependent manner. The P2Y_13_ receptor may represent a negative feedback loop regulating ATP release from osteoblasts. **(2)** Once in the microenvironment, ATP and its breakdown products can activate either P2Y or P2X receptors. P2X7 receptors on osteoclasts precursors are activated by higher concentrations of ATP and are involved in the fusion and multinucleation of osteoclasts: antagonism of P2X7 receptors (α-P2X7) prevents this process. Similarly antagonism of P2Y_12_ receptors (α-P2Y_12_) prevents fusion of osteoclasts as well as their maturation and activity. **(3)** Activation of P2Y_1_ and P2Y_6_ receptors increases osteoclast resorption whilst absence of P2Y_13_ receptors (P2Y_13_R^– / –^) reduces osteoclast number and activity. **(4)** ATP and other nucleotides are potent potentiates of systemic factors such as PTH – providing a mechanism to induce activation of osteoblasts above a threshold attained by systemic factors alone to facilitate focal remodeling as occurs in the case of adaptive remodeling in response to loading or PTH treatment. **(5)** Activation of P2 receptors in bone cells by ATP provides a mechanism to translate the mechanical stimuli engendered in bone into a biological signal. This signal can then be propagated through the network of cells in bone, including osteocytes which have also been shown to release ATP upon mechanical loading.

This review will discuss the most recent developments in the field of purinergic signaling in bone, highlighting work from a recent European Framework 7 funded collaboration called ATPBone, as well as Arthritis Research UK and Bone Research Society funded projects (see **Figure 1** for a summary).

## MODULATION OF ATP RELEASE FROM BONE CELLS

Bone is subjected to a wide array of mechanical stimuli that include fluid shear stress which occurs when the interstitial fluid within the canaliculi of bone flows past the cells of bone in response to movement, substrate strain, and compressive loading (Ehrlich and Lanyon, 2002; Baron, 2003; Klein-Nulend et al., 2005). It is well established that mechanical stimulus applied to cells *in vitro*, even as subtle as fluid movements after a medium change, can increase basal ATP release (Lazarowski et al., 2000). A role for ATP release in the process of mechanotransduction in bone has been suggested for some time now (Turner, 2006), although, as with the original concept of purinergic signaling, it is not yet widely acknowledged or appreciated.

Progress in unraveling the processes behind ATP release from osteoblasts has been advanced with results from a recent study comparing the effects of all of the loading forms experienced by bone including fluid flow, fluid shear stress, substrate strain, and 3D compressive loading (Rumney et al., 2012). ATP release was demonstrated to increase rapidly in response to turbulent fluid flow, with a gradual increase in response to fluid shear stress and substrate strain. A functional consequence of mechanical load-induced ATP release was shown to be the activation of the osteogenic immediate early gene c-*fos*, which was demonstrated to be activated most in response to 3D fluid flow and substrate strain. The results of this study, which was partly funded by a Bone Research Society Barbara Mawer Travelling fellowship, demonstrated that the amount of ATP released in response to mechanical loading varied in a time, direction, and strain-dependent manner and that this may represent a local mechanostat in bone that may influence osteogenesis.

Until recently, research on ATP release from bone cells has focused on osteoblasts, with a few reports on release of ATP from osteocytes (Genetos et al., 2007). A recent report highlights the importance of the voltage-sensitive calcium channel α_2_δ_1_ auxiliary subunit in regulating mechanically induced ATP release in the osteocytic cell line MLO-Y4 (Thompson et al., 2011) offering further insights into the mechanisms behind regulation of ATP release in response to mechanical load.

Exciting new data has also emerged on the release of ATP from osteoclasts. In an oral presentation at the 2011 joint meeting of the Bone Research Society & the British Orthopaedic Research Society, Rumney et al. (2011) demonstrated for the first time that primary human osteoclasts release ATP and that active osteoclasts have an enhanced sensitivity to fluid flow. Until then little, if anything, had been reported on whether osteoclasts were capable of releasing ATP either constitutively or in response to mechanical loading. The concept that osteoclasts may be directly involved in sensing mechanical loading in bone via ATP release is further supported by recent data from Brandao-Burch et al. (2012) reported in this research topic. They provided evidence for the involvement of the mechanosensitive P2X7 purinoceptor in the release of ATP from murine osteoclasts. Taken together, these two reports provide an intriguing new direction of research in bone mechanotransduction.

## RECENT ADVANCES RELATING P2X7 RECEPTOR ACTIVITY AND OSTEOCLAST MULTINUCLEATION

The P2X7 receptor was originally discovered as the P2Z receptor, sensitive to high levels of ATP and thought to function only in the cytotoxic effect of extracellular ATP (Falzoni et al., 1995) – earning the receptor the nickname “the suicide receptor”! Many advances have been made in elucidating the role of this receptor since this initial proposed function (which surely would have evolved it to extinction if only involved in suicide?) including the release of cytokines and more contentiously cell fusion (Lemaire et al., 2011a). Both these proposed functions play key roles in bone homeostasis and accordingly several labs have investigated the role of the P2X7 receptor in bone cells, and more specifically in osteoclast fusion and multinucleation: *in vitro* blockade of the receptor prevents fusion (Gartland et al., 2003a), whilst the P2X7R KO mice maintain the ability to form multinucleated osteoclasts *in vitro* and *in vivo* (Gartland et al., 2003b; Ke et al., 2003; for review see Gartland, 2012).

Multinucleation of osteoclasts is similarly a contentious issue – it probably still remains a debate as to whether multinucleation occurs to increase the efficiency of resorption, possibly via increased transcriptional activity (Boissy et al., 2002), or not (Piper et al., 1992; Lees et al., 2001). Regardless of the reason for multinucleation of osteoclasts, the role of the P2X7 receptor in this process has been strengthened recently. Using a number of different P2X7 receptor antagonists, Agrawal et al. (2010) demonstrated that all but one compound decreased the formation and function of multinucleated TRAP-positive osteoclasts in a concentration-dependent manner. Whilst this study provides further evidence for the involvement of the P2X7 receptor in fusion of osteoclasts, it did not elucidate the mechanism by which P2X7 receptors are involved in fusion. This was however, addressed in another study by Pellegatti et al. (2011) in which they proposed that P2X7 receptors are absolutely required for fusion of osteoclasts due to P2X7 receptor-dependent release of ATP which is then broken down to adenosine with the subsequent activation of adenosine receptors resulting in cell fusion. The role of P2X7 receptor in ATP release-driven cell fusion was also demonstrated to be critical for the induction of multinucleated macrophages by the inflammatory cytokine GM-CSF (Lemaire et al., 2011b). Targeting the P2X7 receptor in diseases with increased osteoclast multinucleation, such as Paget’s disease, may provide new therapeutic options.

## MOUSE MODELS TO DETERMINE FUNCTIONAL CONSEQUENCES OF PURINERGIC SIGNALING IN BONE

Since the first report over 20 years ago that extracellular ATP could modulate intracellular calcium and second messenger signals in bone (Kumagai et al., 1991; Schofl et al., 1992) numerous groups have reported the expression of P2X and P2Y subtypes by the different bone cell types in a variety of species with wide ranging functional consequences (see reviews listed above for specific details). The most recent data to add to the catalog of expression and function profiles has emerged following the bone phenotype analysis of KO mice for P2Y_6_ receptor and the relatively recently discovered P2Y_13_ receptor.

As part of the EU Framework 7 funded project “ATPBone: Fighting osteoporosis by blocking nucleotides: purinergic signaling in bone formation and homeostasis,” the P2Y_6_ and P2Y_13_ receptor KO mice (P2Y_6_R^– / –^ and P2Y_13_R^– / –^) were made available by Bernard Robaye and Jean Marie Boeynaems from the Institute of Interdisciplinary Research in Human and Molecular Biology, Universtité Libre de Bruxelles. Using P2Y_6_R^– / –^ bone marrow derived cells to generate osteoclasts *in vitro*, Orriss et al. (2011) demonstrated a reduction in bone resorption compared to wild-type (WT) osteoclasts. This data complemented their observation that when UDP, the agonist for the P2Y_6_ receptor, was added to mature murine osteoclasts for the final 48 h of culture it significantly increased resorption by up to 60%. In the same study the authors examined the bones of 2-month-old P2Y_6_R^– / –^ mice and found that there was a reduction in the number of osteoclasts on the surface of the endocortical and trabecular bone. One would predict that given the observed reduction in osteoclast resorptive capacity *in vitro* and osteoclast numbers *in vivo* that the P2Y_6_R^– / –^ mice would have a high bone mass phenotype. However, the authors found no significant effect of receptor deletion on the amount or architecture of trabecular bone either in the long bones or vertebrae of the P2Y_6_R^– / –^ mice, yet they did detect a significant increase in cortical bone volume and thickness. The reason behind this effect in a discrete bone compartment is not yet known, and whether the lack of an extreme bone phenotype is due to purinoceptor redundancy or possible apposing effects of P2Y_6_ receptor deletion on osteoblasts remains to be elucidated. The full publication of further studies using adult P2Y_6_R^– / –^ mice under challenged conditions such as mechanical loading (Gupta et al., 2012) may go some way to explain the enigma of these mice.

The P2Y_13_R^– / –^ mouse had a more obvious bone phenotype that was somewhat unsurprising given the previous reports that ADP, the preferred agonist of the P2Y_13_R, is a powerful osteolytic agent (Hoebertz et al., 2001). In a study recently highlighted by the Editor of Molecular Endocrinology as “excellent examples of the relevance of basic science findings to clinical management of endocrine disorders” (DeFranco, 2012), Wang et al. (2012) found that P2Y_13_R^– / –^ mice had a 40% reduction in trabecular bone volume, a 50% reduction in both osteoblasts and osteoclasts on the surface on bone and an overall 50% reduction in bone remodeling *in vivo*. This reduced bone turnover maybe as a consequence of the reduced ratio of the key bone modulating molecules receptor activator of nuclear factor κ-B ligand and osteoprotegerin and reduced downstream RhoA/ROCK I signaling. Furthermore, the reduced bone turnover observed in the P2Y_13_R^– / –^ mice served to protect them from ovariectomy-induced bone loss by up to 65%. These exciting results highlight a potential alternative drug target for the treatment of bone diseases with accelerated bone turnover such as estrogen deficiency-induced osteoporosis.

Indeed, considerable advances have been made in developing pharmaceutical modulators of the P2Y receptors. Clopidogrel (Plavix^®^), a P2Y_12_ antagonist, was the second most sold pharmaceutical drug in the world in 2010. The P2Y_12_ receptor was first noted to be expressed by bone cells by Buckley et al. (2003). Interestingly, recent reports in abstract form have described an osteopetrotic phenotype of the P2Y_12_R^– / –^ mice (Floyd et al., 2008), with delayed osteoclast formation *in vitro* and decreased Rap1 phosphorylation in response to ADP stimulation in P2Y_12_R^– / –^ cells (Su et al., 2011). These abstracts also suggest a role for the P2Y_12_R in pathological bone loss and we eagerly await the full manuscript.

Another recent development in purinergic signaling using mouse models has arisen thanks to Jørgensen and colleagues at the Research Center for Aging and Osteoporosis, Copenhagen University Hospital who have back-crossed the original Glaxo P2X7R^– / –^ mouse on to the BALB/cJ strain (Syberg et al., 2010). The two original P2X7R^– / –^ mouse models (maintained on mixed C56BL/6 backgrounds) have a natural P451L mutation in the cytoplasmic domain of the P2X7 receptor which confers drastically reduced sensitivity to ATP-induced pore formation. While these existing P2X7R^– / –^ strains have similar and contradictory results in many tissues including bone (i.e., the ability to form multinucleated osteoclasts *in vivo* and *in vitro* and an altered bone phenotype, respectively) there have been many reports that support an important role for the P2X7 receptor in bone (Gartland, 2012). Therefore, analysis of this new P2X7R^– / –^ BALB/cJ strain may offer further new insights into the role of the P2X7R without the possible confounding contribution of the natural P451L mutation. Initial results have demonstrated that these mice also maintain the capability to form multinucleated osteoclasts both *in vivo* and *in vitro* (Gartland, 2012). In addition, when examining the ability of different aged mice to form osteoclasts *in vitro* it appears that formation and function is enhanced in young and developing mice, but that in older mice this increased resorptive capacity of the P2X7R^– / –^ osteoclasts was not as apparent (Agrawal et al., 2012). Again, we look forward to the full report of the bone phenotype of this strain and the contribution of this receptor to osteoclastogenesis.

## PURINERGIC SIGNALING AND BONE DISEASE

Exciting progress has also been made in establishing a role for purinergic signaling in maintenance of healthy bone via studies involving human patient data. The first set of studies involve the gene for the P2X7 receptor, *P2XR7,* which is known to be highly polymorphic with over 26 non-synonymous single nucleotide polymorphisms (SNPs) listed on the NCBI database (Build 131), six of which have been previously described as resulting in either loss or gain of P2X7 receptor function. An initial report from the lab of Jørgensen reported that two SNPs in the P2X7 receptor gene (*P2XR7*) were associated with an increased risk of fracture (Ohlendorff et al., 2007). Since then new SNPs have been found and it has been shown that heterozygote combinations of various SNPs result in an enhanced effect on the receptor function than any individual homozygote SNP. Given these observations further analysis of the role of other *P2XR7 *SNPs as well as combinations of SNPs in bone and indicators of osteoporosis, specifically bone mineral density (BMD), bone loss, and fracture was clearly warranted.

We have recently published the results of our study using women from the Aberdeen Prospective Osteoporosis Screening Study (APOSS) which revealed that a single SNP in the *P2XR7*, the c.946A (p.Arg307Gln), was significantly associated with low BMD at the lumbar spine of women aged 45–54 years and at a follow-up visit between 7 and 9 years later. Further analysis showed that when women with one or more loss-of-function SNP were grouped together they had almost a 10-fold increase in the amount of bone loss per year compared to women who were WT at these positions (Gartland et al., 2012b). This was the first description that a *P2XR7 *polymorphism is associated with BMD, a key determinant of vertebral fracture risk, in post-menopausal women. The involvement of the *P2XR7* in regulating human bone mass was further highlighted with a complimentary article from our ATPBone collaborators (Jørgensen et al., 2012a). By genotyping women from the Danish Osteoporosis Prevention Study (DOPS) cohort for the same functional *P2XR7* SNPs they confirmed the p.Arg307Gln SNP as being important to bone health – as women with this SNP had 40% increased bone loss. They also confirmed the effect of multiple SNPs on bone mass – they found a clear association between the low-risk (high-P2X7 function) alleles and a low rate of bone loss and conversely, high-risk (reduced P2X7 function) alleles were associated with a high rate of bone loss. In addition, they found a gain-of-function SNP was associated with lower vertebral fracture incidence 10 years after the menopause. Taken together, these two independent studies provide strong and compelling evidence that the *P2RX7* is involved in the regulation of bone mass and fracture risk, and may in the future, represent an early diagnostic tool for the management of osteoporosis.

In addition to being implicated in osteoporosis, polymorphisms in the *P2XR7* have been suggested to be involved in other musculoskeletal complications including aseptic hip loosening and rheumatoid arthritis (RA). A Czech group reported that the loss-of-function *P2RX7 *SNPs are overrepresented among patients with total hip arthroplasty (THA) whilst the p.Arg307Gln allele was associated with greater cumulative hazard of THA revision (Mrazek et al., 2010), although the authors acknowledged the limitations of their under-powered study and suggested that replication is needed. Similarly, a report using a small cohort from the Omani Arab population is suggestive that polymorphism at position c.1068 and c.1513 in the P2X7R gene might contribute to the pathogenesis of RA as they were over-represented in the RA group and significantly associated with the presence of rheumatoid factor and anti-MCV autoantibody in RA patients, respectively. Whereas neither the loss-of-function c.1096C > G (p.Thr357Ser) SNP or the gain-of-function c.489C > T (p.His155Tyr) SNP appeared to be a susceptibility gene locus for the development of RA (Al-Shukaili et al., 2012). Both the c.946A (p.Arg307Gln) and c.1729A (p.Ile568Asn) SNPs were not detected in the Omani Arab population, probably due to low numbers (MAF 0.01 and 0.03, respectively, in a Caucasian population). Again, the authors acknowledged that further clarification and replication in a larger cohort was warranted to confirm their findings.

Clearly, further replication of the above observations in RA and aseptic loosening are warranted. As are further analysis of large cohorts such as DOPS and APOSS for SNPs in other purinergic receptors such as the P2Y_2_ receptor, which has a role in bone homeostasis and is also known to have numerous functional polymorphisms associated with other human diseases (Wesselius et al., 2011), are eagerly anticipated.

As stated earlier, purinergic signaling is the most primitive and ubiquitous cell-to-cell signaling system that exists, and one might worry that any drug developed specifically to target one particular receptor for one particular disease may have effects in other systems. The P2Y_12_ receptor is known to play an important role in platelet activation – and P2Y_12_ antagonists have proven therapeutic value in the treatment and prevention of coronary artery disease. As mentioned earlier, the P2Y_12_ receptor has previously been identified as being expressed on human osteoblasts (Buckley et al., 2003) and an osteopetrotic phenotype of the P2Y_12_R^– / –^ mice has been reported (Floyd et al., 2008), although the physiological consequence of P2Y_12_ receptor activation or inhibition in human bone is not known.

Taking these previous reports into consideration, Vestergaard et al. (2011) investigated the use of platelet inhibitors including clopidogrel. In this study, they found that the inhibitors dipyridamole and acetylsalicylic acid were associated with increased fracture, whereas clopidogrel was not. Even though this was a large scale study where all patients sustaining a fracture during the year of 2000 from the Danish population were included (approximately 124,655 cases from 5.3 million individuals), the authors postulated that the lack of any association of fracture risk to clopidogrel was due to the relatively short duration of exposure the patients would have had as the drug was only licensed in July 1998. Given that the number of defined daily doses (DDD) of clopidogrel sold increased by a factor of 30 from 103,000 in 1999 to 3.4 million in 2003 in Denmark alone additional studies in newer data sets were clearly warranted. To that end, Jørgensen et al. (2012b) again used the Danish population to identify patients who were prescribed clopidogrel during the years 1996–2008 (*n* = 77,503) and three non-users, matched for age and gender (*n* = 232,510), for each clopidogrel-treated subject to test for an association between clopidogrel use and fracture incidence. Using this data set they found a biphasic response to clopidogrel use, whereby those patients who had low exposure to treatment with clopidogrel (<0.01 DDD) had a lower risk of fracture than non-users, whilst the remaining patients prescribed clopidogrel had increased overall fracture risk and increased risk of osteoporotic fractures, especially in subjects with a treatment duration of more than 1 year.

What is behind these associations has yet to be elucidated. It could be as a consequence of low-grade inflammation associated with atherosclerosis: inflammation is often accompanied by bone loss and subsequently osteoporosis, thus the inflammation related to cardiovascular disease could be a contributing factor to the bone loss in the clopidogrel-treated group.

Direct antagonism of P2Y_12_ receptor expressed by both osteoblasts and osteoclasts could also be a contributing factor. Antagonism of the P2Y_12_ receptor on both cell types would disrupt the balance of bone homeostasis modulating both bone formation and resorption. This has recently been confirmed by Syberg et al. (2012) who demonstrated that clopidogrel treatment of osteoblast and osteoclast cultures *in vitro* resulted in reduced osteoblast activity and mineralization, an increase in adipocytes and reduced osteoclast formation, viability, and resorptive activity. One might speculate that the *in vitro* observations were due to a non-specific effect of clopidogrel, which is a pro-drug that is rapidly metabolized in the liver to form the active metabolite; whether this occurs in these cultures is undetermined. The authors address this in their manuscript by providing supporting *in vitro *evidence for a direct effect in the form of alterations in cAMP levels and mRNA for key osteoblastic genes in osteoblasts following clopidogrel treatment. However, they acknowledge that off target, non-P2Y_12_ receptor-mediated effects on osteoblasts *in vitro* cannot be fully discounted. They then provide further *in vivo* evidence that clopidogrel has a detrimental effect on bone by comparing the bone parameters of ovariectomized mice with ovariectomized mice treated daily for 4 weeks with clopidogrel. Clopidogrel-treated mice had exaggerated effects of ovariectomy including significantly increased bone resorption and decreased bone formation markers, as well as enhanced bone loss as evidenced by significantly larger reductions in BMD, trabecular bone volume, and trabecular number. Clearly, further studies into the exact mechanism of action of clopidogrel, and the newer and more potent anti-platelet drugs such as prasugrel and ticagrelor, on bone metabolism are both timely and necessary.

Finally, the role of purinergic signaling in cancer is gradually emerging (Deli and Csernoch, 2008, but the role of ATP and P2 receptors in bone cancer and cancer-induced bone disease (CIBD) still relatively unexplored. The P2X7 receptor has been implicated in many cancer types and its antagonism proposed as a potential treatment (Roger and Pelegrin, 2011). Given its role in bone cells one would speculate that it would be involved in bone cancer or CIBD. To date, only one study has investigated purinergic signaling in bone cancer, specifically the P2X7 receptor in bone cancer pain. In this study, Hansen et al. (2011) injected 4T1 cancer cells directly into the femur of the mice and then measured pain-related behaviors and also bone destruction and tumor burden. They demonstrated that P2X7R-deficient mice were still susceptible to bone cancer pain and compared to cancer-bearing WT mice had an earlier onset of pain-related behaviors. There was no apparent difference in the bone destruction or tumor burden between WT and P2X7R-deficient mice, although the authors note that due to the advanced stage of cancer there was not a reliable measure of tumor burden in their model. They suggest that differences between the two genotypes could not be ruled out and experiments exploring earlier time points to fully elucidate the role of the P2X7 receptor in bone cancer are warranted.

## SUMMARY

Clearly, we have come a long way in purinergic signaling in bone since the first description almost 20 years ago and we have made significant advances in the past few years thanks to some great collaboration. The availability of more KO (and transgenic) models as well as the development and use of specific antagonists for other conditions will undoubtedly help us to elucidate further the exact roles purinergic signaling pathways have to play in bone homeostasis, both in health and disease.

## Conflict of Interest Statement:

The authors declare that the research was conducted in the absence of any commercial or financial relationships that could be construed as a potential conflict of interest.
